# Plectin Downregulation Inhibits Migration and Suppresses Epithelial Mesenchymal Transformation of Hepatocellular Carcinoma Cells via ERK1/2 Signaling

**DOI:** 10.3390/ijms24010073

**Published:** 2022-12-21

**Authors:** Rushuang Xu, Shan He, Di Ma, Rui Liang, Qing Luo, Guanbin Song

**Affiliations:** Key Laboratory of Biorheological Science and Technology, Ministry of Education, College of Bioengineering, Chongqing University, Chongqing 400030, China

**Keywords:** plectin, hepatocellular carcinoma, migration, epithelial mesenchymal transformation, ERK1/2

## Abstract

Plectin, as a cytoskeleton-related protein, is involved in various physiological and pathological processes of many cell types. Studies have found that plectin affects cancer cell invasion and metastasis, but the exact mechanism is not fully understood. In this study, we aim to investigate the role of plectin in the migration of hepatocellular carcinoma (HCC) cells and explore its relevant molecular mechanism. Herein, we found that the expression of plectin in HCC tissue and cells was significantly increased compared with normal liver tissue and cells. After downregulation of plectin, the migration ability of HCC cells was significantly lower than that of the control group. Moreover, the expression of E-cadherin was upregulated and the expression of N-cadherin and vimentin was downregulated, suggesting that plectin downregulation suppresses epithelial mesenchymal transformation (EMT) of HCC cells. Mechanically, we found that plectin downregulation repressed the extracellular signal-regulated kinase 1/2 (ERK1/2) phosphorylation. Activation of ERK1/2 recovered the plectin downregulation-inhibited migration and EMT of HCC cells. Taken together, our results demonstrate that downregulation of plectin inhibits HCC cell migration and EMT through ERK1/2 signaling, which provides a novel prognostic biomarker and potential therapeutic target for HCC.

## 1. Introduction

Hepatocellular carcinoma (HCC) is one of the most frequent primary cancers that seriously endanger human health. According to global statistics, in 2020 HCC was the sixth most commonly diagnosed cancer and the third leading cause of cancer-related death worldwide. In Asia (especially China), the incidence and mortality of HCC are also high-ranking [1]. HCC patients have poor prognosis, high recurrence and metastasis rates and a low five-year survival rate; invasion and metastasis are also the most prominent biological characteristics of HCC [2]. The metastasis of HCC is caused by the action of multiple factors and genes, which is an essential reason for the death of cancer patients [3,4]. Therefore, more reliable biomarkers for understanding HCC metastasis mechanisms need to be developed urgently.

Plectin is a high molecular weight, multidomain protein that has attracted much attention in recent years, and it was initially characterized as a scaffolding protein [5,6]. Plectin links the three cytoskeletons to maintain the integrity of the cytoskeleton and cell morphology, and also is part of desmosomes or hemidesmosomes that participate in intercellular or cell-basal junctions [7,8]. In addition, plectin is an important regulator of cell activities affecting cell migration, proliferation and regulation of mitochondrial function [9,10,11]. Given the significance of plectin in cytophysiology, its clinical relevance and potential role in cancers deserve to be investigated.

Recently, the importance of plectin in tumorigenesis has become increasingly recognized. Abnormal expression of plectin has been found to be associated with the malignant phenotype of colorectal cancer, pancreatic cancer and head and neck squamous carcinoma [12,13,14]. Plectin is upregulated in many cancers and promotes their metastasis; thus, plectin is also used as a biomarker for some cancers [13]. Therefore, the effect of plectin on the occurrence and development of HCC deserves our attention. In addition, researchers have linked aberrant expression of plectin to cancer cell migration and invasion, but the underlying molecular mechanisms remain largely unaddressed. In this study, we aim to investigate the effects of plectin on HCC cell migration and unravel the possible molecular mechanism.

## 2. Results

### 2.1. Plectin Is Significantly Upregulated in HCC Tissue and Cells, and Promotes HCC Cell Migration

**Figure 1:**
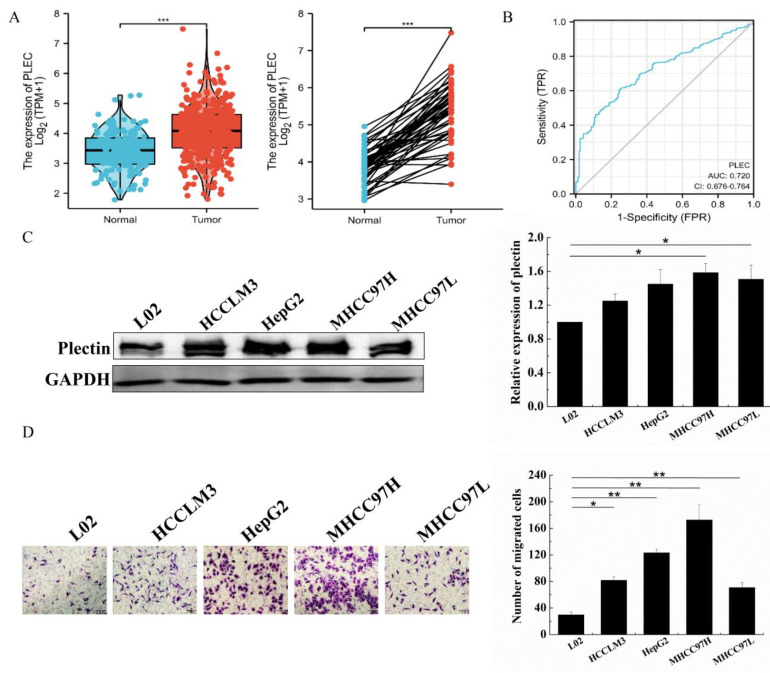
Plectin expression is upregulated in HCC tissue/cells and promotes HCC cell migration. (**A**) Plectin mRNA expression in HCC and normal tissue with unpaired samples (left) and paired samples (right) from the UALCAN database. (**B**) ROC curve of plectin in HCC. (**C**) Western blot detection of plectin protein expression in a panel of HCC cell lines including HCCLM3, HepG2, MHCC97H and MHCC97L cells and human liver cell line L02. (**D**) Transwell assays for migration ability evaluation in a panel of HCC cell lines including HCCLM3, HepG2, MHCC97H and MHCC97L cells and human liver cell line L02. The number of cells were counted and averaged (scale bar, 100 µm). GAPDH: glyceraldehyde-3-phosphate dehydrogenase. Data are presented as mean with SD; *n* = 3, * *p* < 0.05; ** *p* < 0.01; *** *p* < 0.001.

To detect plectin mRNA expression in HCC cells, we analyzed plectin expression in TCGA. Analysis of unpaired data showed that the plectin mRNA expression level was significantly higher in HCC tissue (*n* = 371) than that in normal tissue (*n* = 160), and paired data analysis also showed that plectin mRNA expression levels were significantly higher in HCC tissue than those in normal tissue (*n* = 50) (Figure 1A). These results indicated that plectin mRNA expression was upregulated in HCC tissue. In order to realize the diagnostic value of plectin in differentiating HCC samples from normal samples, receiver operating characteristic (ROC) curve analysis was performed. ROC curve analysis showed that the area under the plectin’s curve (AUC) was 0.720 (Figure 1B), indicating that plectin possessed favorable diagnostic value. We also analyzed the expression of plectin in four HCC cell lines (HCCLM3, HepG2, MHCC97H and MHCC97L cells) and human liver cell line L02. Western blot analysis further confirmed plectin expression was higher than that in liver cell L02 (Figure 1C). Collectively, these results suggest that plectin is upregulated in HCC tissue and cells. Thus, it may play an important role in HCC development.

To investigate the effects of plectin on HCC cell migration, we detected the migration ability in L02 and HCC cells, and the migration ability of various HCC cells was indeed significantly higher than that of L02 (Figure 1D). Based on the results of plectin expression and migration in these HCC cells, MHCC97H and MHCC97L cells were employed for the follow-up experiments.

### 2.2. Plectin Downregulation Inhibits HCC Cell Migration and Epithelial Mesenchymal Transformation

**Figure 2:**
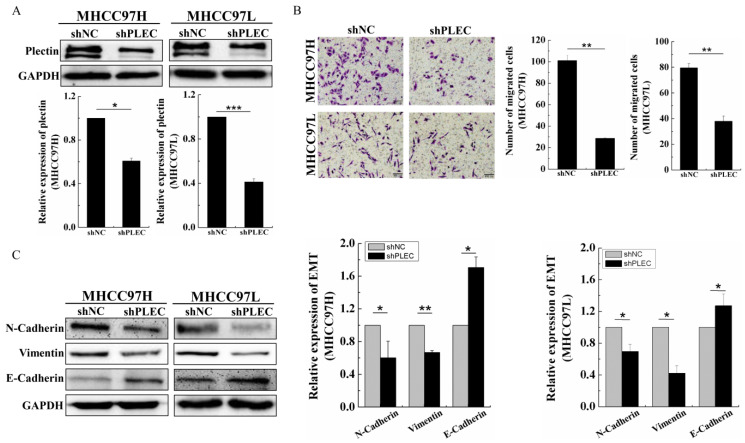
Plectin downregulation inhibits HCC cell migration and EMT. (**A**) Western blot detection of plectin protein level in cells transfected with the shNC and shPLEC and quantification data of Western blot detection of plectin protein level in cells transfected with the shNC and shPLEC. (**B**) Transwell assays for migration ability evaluation in cells transfected with the shNC and shPLEC. The number of cells were counted and averaged (scale bar, 100 µm). (**C**) Western blot detection of N-cadherin, vimentin and E-cadherin in cells transfected with the shNC and shPLEC and quantification data of Western blot detection of N-cadherin, vimentin and E-cadherin protein level in cells transfected with the shNC and shPLEC. GAPDH: glyceraldehyde-3-phosphate dehydrogenase. Data are presented as mean with SD; *n* = 3, * *p* < 0.05; ** *p* < 0.01; *** *p* < 0.001.

Considering the high expression of plectin in HCC tissue and cells, we knocked down plectin with shRNAs in MHCC97H and MHCC97L cells. Western blot analysis showed significant downregulation of plectin in MHCC97H and MHCC97L cells upon transfection of PLEC-shRNAs (shPLEC) as compared with scramble transfected cells (shNC) (Figure 2A). Subsequently, the transwell assay showed that shPLEC transfection led to a significant decrease in the migration number in MHCC97H and MHCC97L cells compared with shNC transfection cells (Figure 2B). These results suggest that plectin is essential for HCC cell migration.

Epithelial mesenchymal transformation (EMT) plays an important role in the invasion and metastasis of cancer [15]. Therefore, the epithelial marker E-cadherin and the mesenchymal markers N-cadherin and vimentin were analyzed to examine whether plectin affects EMT progress in MHCC97H and MHCC97L cells. We found that the downregulation of plectin led to increased protein levels of E-cadherin and decreased protein levels of N-cadherin and vimentin compared with the shNC cells (Figure 2C). Collectively, these results suggest that plectin downregulation inhibits HCC cell migration and EMT.

### 2.3. Plectin Downregulation Inhibits HCC Cell Migration and EMT via ERK1/2 Signaling

**Figure 3:**
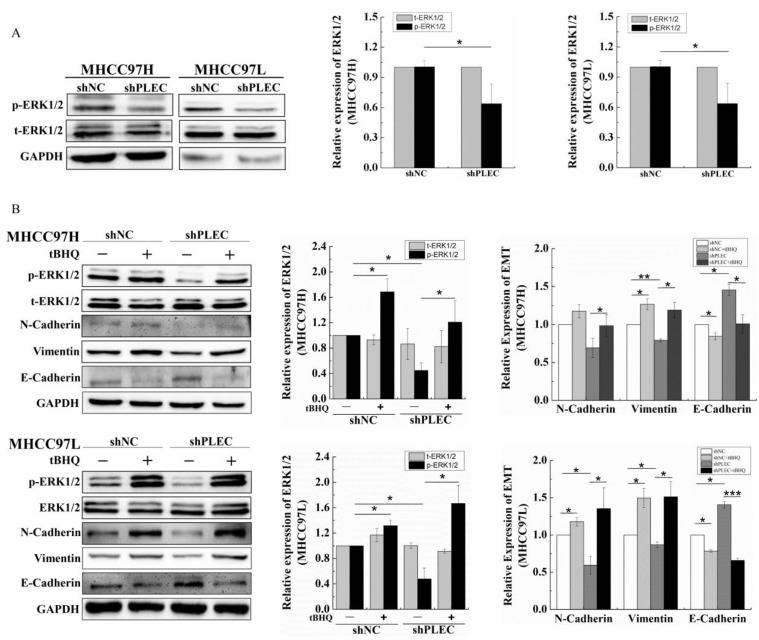
Activation of ERK1/2 recovered the plectin downregulation-inhibited EMT of HCC cells. (**A**) Western blot detection of p-ERK1/2 and t-ERK1/2 protein expression in cells transfected with shNC and shPLEC and quantification data of Western blot detection of p-ERK1/2 and t-ERK1/2 protein levels in cells transfected with the shNC and shPLEC. (**B**) Western blot detection of the p-ERK1/2, t-ERK1/2, N-cadherin, vimentin and E-cadherin protein levels after tBHQ treatment in cells transfected with shNC and shPLEC and quantification data of Western blot detection of p-ERK1/2, t-ERK1/2, N-cadherin, vimentin and E-cadherin protein levels after tBHQ treatment in cells transfected with shNC and shPLEC. p-ERK1/2: phosphorylation of extracellular regulated protein kinases 1/2; t-ERK1/2: total extracellular regulated protein kinases 1/2; tBHQ: tert-butylhydroquinone; GAPDH: glyceraldehyde-3-phosphate dehydrogenase. Data are presented as mean with SD; *n* = 3, * *p* < 0.05; ** *p* < 0.01; *** *p* < 0.001.

**Figure 4:**
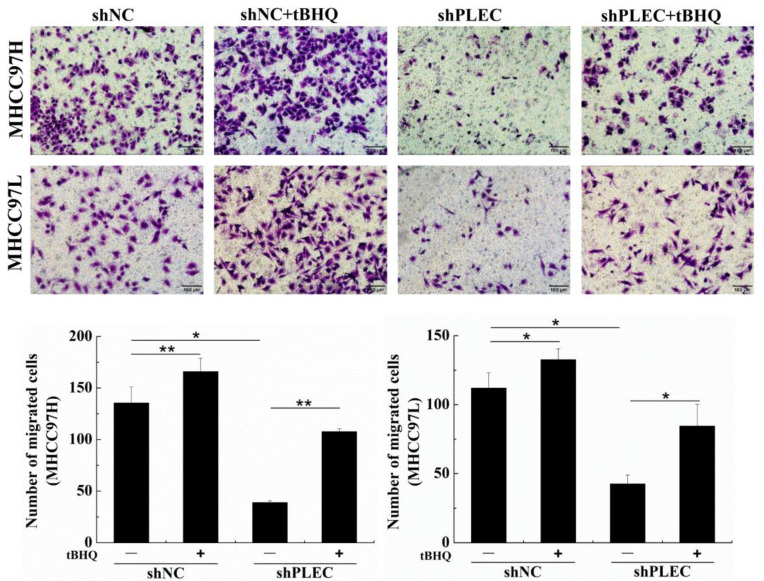
Activation of ERK1/2 recovered the plectin downregulation-inhibited migration of HCC cells. Transwell assays for migration ability evaluation and quantification data of migrated number after tBHQ treatment in cells transfected with shNC and shPLEC (scale bar, 100 μm). tBHQ: tert-butylhydroquinone. Data are presented as mean with SD; *n* = 3, * *p* < 0.05; ** *p* < 0.01.

Previous studies have reported that phosphorylation of extracellular regulated protein kinases 1/2 (p-ERK1/2) plays a critical role in cancer cell migration [16,17]. Other studies have also showed that the upregulation of p-ERK 1/2 affects tumor invasion and metastasis by inducing EMT or MMP [18,19,20,21]. Therefore, to confirm the potential role of ERK1/2 in plectin’s effect on HCC cell migration and EMT progress, we detected p-ERK1/2 protein expression after plectin depletion by Western blot. The results showed that p-ERK1/2 was significantly decreased in shPLEC transfected MHCC97H and MHCC97L cells compared with the shNC group (Figure 3A). Tert-butylhydroquinone (tBHQ) is an ERK1/2 activator, and the formation of oxidative metabolites derived from tBHQ is responsible for the ERK1/2 activation [22]. To further clarify the role of p-ERK1/2 in plectin regulation of HCC cell migration, we treated shNC and shPLEC cells with tBHQ, and found that tBHQ treatment did not change the expression of total ERK1/2 (t-ERK1/2) but restored the expression of p-ERK1/2 in MHCC97H and MHCC97L cells. Meanwhile, tBHQ treatment recovered the EMT progress in shNC and shPLEC cells (Figure 3B). Moreover, the transwell assay showed that the migration ability of the shNC and shPLEC cells treated with tBHQ increased dramatically (Figure 4). These results demonstrate that ERK1/2 activation plays a crucial role in HCC cell migration and the EMT process affected by plectin expression.

## 3. Discussion

In the present study, we examined the role of plectin in regulating HCC cell EMT and migration. We found that plectin expression is upregulated in HCC tissue and cells. Downregulation of plectin inhibits migration and suppresses EMT of HCC cells. Furthermore, we found that plectin promotes HCC cell migration and the EMT process depends on ERK1/2 signaling.

More studies have shown that cells are intricately connected to the external environment through their cytoskeleton, and the tension generated by a contracting cytoskeleton can be used to sense the mechanical properties of the extracellular matrix [15,23]. Additionally, one other study has reported that plectin can affect the arrangement of intermediate filaments cytoskeleton and thus affect cell morphology and biological behavior [24]. Therefore, this indirectly confirm the conclusion that plectin is an important mechanosensor [25]. Plectin could transmit signals to the nucleus through cytoskeleton, focal adhesion, desmosome, nuclear membrane and others [26,27], and finally give rise to the corresponding biological behavior. All this suggests that plectin probably plays an important role in mechanotransduction in response to the change in the mechanical environment.

As we know, the process of liver lesions is typical of mechanical microenvironment changes. Chronic liver diseases leading to HCC are associated with ECM over-production, and the stiffness in liver cancer tissue is approximately 10 times that of normal liver tissue [28]. Moreover, our previous study has demonstrated that the different regions of HCC tissue exhibit mechanical heterogeneity, and the distribution of liver cancer stem cells correlates with the mechanical heterogeneity of liver cancer tissue [29]. These results indicate that the process from liver lesions to HCC involves complex mechanical microenvironments, closely related to changes in cell mechanical properties. In this study, we found that plectin expression in HCC tissue and cells was significantly higher than that in normal liver tissue and cells. Interestingly, plectin downregulation inhibits HCC cell migration and suppresses EMT. These results suggest that plectin may be a mechanosensor in liver lesions. Our future work will address the potential role of this mechanosensor in mechanotransduction on the occurrence and development of HCC.

Increasing evidence has demonstrated that plectin affects cell migration and invasion and metastasis of neoplasm, but the molecular mechanisms involved are not fully understood. In head and neck squamous cancer cells and colon cancer cells, plectin promoted the migration and invasion of these cells through the activation of ERK1/2 [12,30]. Our results in HCC cells are consistent with these findings. We also identified ERK1/2 as an important regulator of downstream EMT molecular expression and migration. In melanoma cells, the downregulation of plectin activated ERK1/2, which in turn promoted the migration of the cells [31]. Based on the above studies, plectin expression could contribute to the downstream ERK1/2 activity in different cells. Interestingly, one study has shown that ERK1/2 activity responds to mechanical stimulation, thereby affecting cell migration, proliferation and other biological behavior [32]. It may implicate that ERK1/2 is a crucial signal molecule responding to mechanochemical stimuli in plectin-mediated cell migration.

EMT drives cancer cell invasion and leads to metastasis. EMT-related transcription factors such as ZEB1, snail, and twist are the important links that regulate the expression of epithelial genes and thus affect the invasion and metastasis of cancer cells [33]. Our experiments have demonstrated that plectin can affect the expression of epithelial and mesenchymal proteins. Therefore, it would be interesting to study whether plectin affects transcription factors related to repressor epithelial gene expression, which would further indicate the relationship between plectin and EMT.

**Figure 5:**
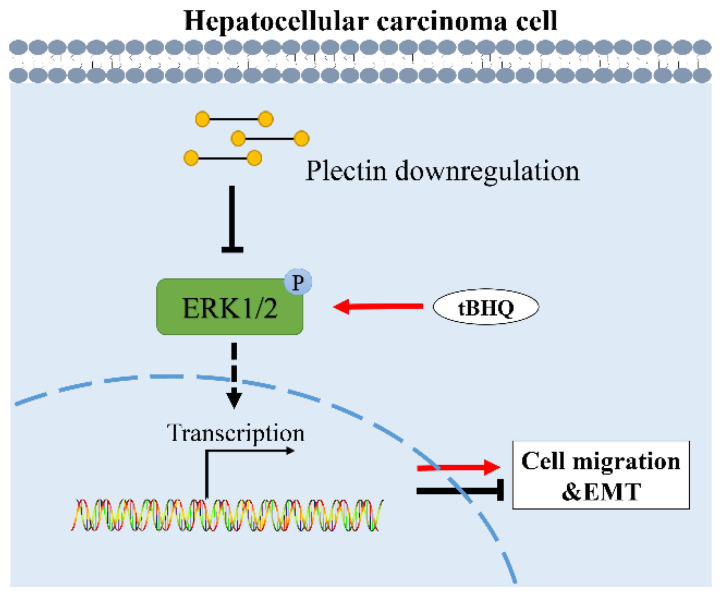
Proposed schematic diagram of the regulation of HCC cell migration and EMT by plectin downregulation. Downregulation of plectin decreases the expression of p-ERK1/2, then inhibits the EMT process and migration ability of HCC cells. tBHQ treatment recovers p-ERK1/2 protein expression and recovers the EMT process and migration ability of HCC cells caused by plectin downregulation. This confirms that downregulation of plectin inhibits HCC cell migration and EMT through ERK1/2 signaling. EMT: epithelial mesenchymal transformation; p-ERK1/2: phosphorylation of extracellular regulated protein kinases 1/2; tBHQ: tert-butylhydroquinone.

In conclusion, in the present work, we find that plectin is upregulated in HCC tissue and cells, and plectin downregulation inhibits migration and suppresses EMT of HCC cells. Furthermore, we demonstrate that plectin downregulation-affected migration and EMT in HCC cells depend on the ERK1/2 signaling pathway. A proposed schematic diagram that summarizes the molecular mechanism of plectin regulating HCC cell migration and EMT is shown in Figure 5. Altogether, our data identify the important role of plectin in HCC cell migration, which provides a novel prognostic biomarker and potential therapeutic target for HCC.

## 4. Materials and Methods

### 4.1. Bioinformatics Analysis

The transcriptional expression data and clinical information of plectin in human liver and HCC tissue were downloaded from the Cancer Genome Atlas (TCGA) website. The RNA-seq gene expression data of workflow fragments per kilobase of exon model per million mapped fragments (FPKM) were converted into transcripts per kilobase of exon model per million mapped reads (TPM) format and log2 conversion was performed for subsequent analysis [34]. All data were obtained from the TCGA database and did not require ethics committee approval.

### 4.2. Cell Culture

The human liver cell line L02 and the HCC cell lines MHCC97H, MHCC97L, HCCLM3 and HepG2 cells were bought from the Liver Cancer Institute, Zhongshan Hospital, Fudan University (Shanghai, China). Cells were maintained in high-glucose Dulbecco’s Modified Eagle’s Medium (DMEM, Biological Industries, Kibbutz Beit Haemek, Israel) supplemented with 10% fetal bovine serum (FBS, Biological Industries), 100 μg/mL streptomycin and 100 U/mL penicillin (Hyclone, Logan, UT) at 37 °C in a humidified atmosphere with 5% CO_2_. When cells grew to 80–90% confluence, a solution of 0.25% trypsin-0.02% EDTA was used to digest cells and cells were sub-cultured at a density of 2.5 × 10^3^ cells/cm^2^.

### 4.3. Protein Extraction and Western Blot

The total protein of the MHCC97H and MHCC97L cells was extracted on ice using a RIPA lysis buffer (Beyotime, Shanghai, China) supplemented with 1% protease inhibitor (Beyotime, Shanghai, China) and 2% phosphatase inhibitors. Protein concentrations of the lysates were measured with a BCA protein assay kit (Beyotime, Shanghai, China). The lysates mixed with a 5× loading buffer were boiled for 10 min at 100 °C for denaturation. Then, 30 µg of each protein sample was separated with 6% or 10% SDS-PAGE gel and was electroblotted onto 0.45 μm PVDF membranes (Millipore, Billerica, MA, USA). The membranes were blocked for 1 h at room temperature with 5% non-fat milk in tris-buffered saline with 0.05% Tween-20 (TBST). Then, the membranes were incubated with primary antibodies including plectin (ab32528, Abcam, UK), N-Cadherin (ab76011, Abcam, UK), vimentin (ab92547, Abcam, UK), E-Cadherin (ab40772, Abcam, UK), t-ERK1/2 (ab184699, Abcam, UK), p-ERK1/2 (4370T, Cell Signaling Technology, Danvers, MA, USA) and GAPDH (bsm-33033M, Bioss, China) diluted in primary antibody diluents (Beyotime, Shanghai, China) overnight at 4 °C. Next, the membrane was washed 4 times with TBST for 5 min each time and incubated with an appropriate secondary antibody (ZENBIO, Chengdu, China) with 5% non-fat milk for 1 h at room temperature and then washed 4 times with TBST for 5 min each time, the protein expression was then visualized using an ECL blotting analysis system (Bio-OI, Guangzhou, China).

### 4.4. Cell Migration Assay

A transwell assay was employed to evaluate cell migration. Briefly, the cells in the logarithmic growth phase were taken for normal digestion and then resuspended in serum-free medium to adjust the density to 2 × 10^5^ cells/mL; 100 μL of the cell suspension was seeded in the upper compartment of the chamber (8 μm pores; Millipore, MA, USA) and 600 μL of medium with 10% FBS was added to the lower compartment as a chemoattractant. After incubating for 24 h in an incubator with the conditions of 37 °C and 5% CO_2_, the inner surface of the upper filter was scrubbed with a cotton-tipped swab to remove non-migrating cells, and it was fixed with 4% paraformaldehyde for 20 min at room temperature. The chamber was blotted with 4% paraformaldehyde, and the chamber was placed into 0.1% crystal violet staining solution for 30 min at room temperature. The chamber was soaked in double steaming water and dried. Cells on the underside of filters in the visual field were randomly observed under a microscope and photographed. The number of cells in each field were counted and averaged.

### 4.5. Virus Production and Transfection

Plasmid systems including pLVX-EGFP-PLEC (Unibio, Hunan, China), psPAX2 (Invitrogen, CA, USA) and pMD2.G (Invitrogen, CA, USA) were used for lentivirus infection. Plasmids were mixed and transiently transfected into 293T cells (the Liver Cancer Institute of Fudan University, Shanghai, China), gently mixed and then placed into an incubator for 48 h. Then, the lentivirus, which was unpurified disease venom, was collected and stored in a refrigerator at −80 °C. For lentivirus infection, about 1 × 10^4^ cells were cultured in 12-well plates, and the lentivirus was then added. The HCC cells were infected with the lentivirus in the presence of 8 μg/mL polybrene (Sigma Aldrich, MO, USA). After 48 h cultivation, the cells’ fluorescence intensity was detected by an inverted fluorescence microscope. Then, 2 μg/mL puromycin was added to select stable clones for one week. Finally, the stable expression of the target gene was detected by Western blot. The target sequences used for shRNA gene-silencing plasmids were as follows: shPLEC-1: GCCUCUUCAAUGCCAUCAUTT AUGAUGGCAUUGAAGAGGCTT; shPLEC-2: GCCAGUACAUCAAGUUCAUTT AUGAACUUGAUGUACUGGCTT.

### 4.6. Reagent Treatment

To activate the ERK pathway, HCC cells were treated with tert-butylhydroquinone (50 μM, HY-100489, MCE) for 24 h.

### 4.7. Statistical Analysis

Statistical analysis was performed using Origin 8.5 software. All data were expressed as the mean ± SD. Independent experiments were repeated at least 3 times for each assay. *p* values were analyzed by one-way analysis of variance (ANOVA) followed by a two-tailed Student’s *t*-test. When *p* < 0.05, the data between two groups was considered to have statistically significant differences.

## Data Availability

All data are contained within the article.
